# Traditional uses, phytochemistry, pharmacology, toxicity and clinical application of traditional Chinese medicine *Cynoglossum amabile*: a review

**DOI:** 10.3389/fphar.2024.1325283

**Published:** 2024-04-09

**Authors:** Yanxiao Fan, Miaomiao Wang, Qing Zhang, Shuqi Ouyang, Wenhui Mao, Congli Xu, Min Wang, Chunlin Long

**Affiliations:** ^1^ Key Laboratory of Ecology and Environment in Minority Areas (Minzu University of China), National Ethnic Affairs Commission, Beijing, China; ^2^ College of Life and Environmental Sciences, Minzu University of China, Beijing, China; ^3^ Xianggelila Bureau of Forestry and Grassland, Beijing, China; ^4^ Baoshan Administrative of Gaoligongshan National Nature Reserve, Baoshan, China; ^5^ College of Chemistry and Materials Engineering, Beijing Technology and Business University, Beijing, China; ^6^ BTBU-TANGYI Innovation Center for the Evaluation of the Safety and Efficacy of Bioengineering Raw Materials, Beijing, China; ^7^ Key Laboratory of Ethnomedicine (Minzu University of China), Ministry of Education, Beijing, China; ^8^ Institute of National Security Studies, Minzu University of China, Beijing, China

**Keywords:** *Cynoglossum amabile*, ethnomedicine, traditional utilization, pyrrolizidine alkaloids, pharmacology, hepatotoxicity

## Abstract

Cynoglossum amabile, a member of the Boraginaceae family, is a well-known traditional Chinese medicine and ethnomedicine known as Daotihu. Despite several studies confirming the presence of bioactive pyrrolizidine alkaloids such as amabiline, ambelline, echinatine, europine, and others in *C. amabile*, there has been no comprehensive review of its traditional uses, phytochemistry, and pharmacology thus far. This review was conducted by thoroughly examining the literature and analyzing network databases. It covers various aspects of *C. amabile*, including botanical characteristics, geographical distribution, traditional applications, phytochemistry, pharmacological activities, toxicology, and clinical applications. The results have shown that *C. amabile* has been traditionally used for medicinal, edible, and ornamental purposes in China for many centuries. The whole plant, root, and leaf of *C. amabile* are used by different ethnic groups, such as Lisu, Bai, Naxi, Yi, Jinuo, and Han, to treat malaria, hepatitis, dysentery, leucorrhea, tuberculosis cough, fracture, joint dislocation, trauma bleeding, and skin carbuncle abscess. A total of 47 chemical components, including alkaloids (pyrrolizidine alkaloids, PAs), sterols, organic acids, and saccharides, were isolated from *C. amabile*. Pharmacological studies show that the chemical extracts of *C. amabile* possess various biological activities, such as anti-inflammatory, anti-tumor, anti-microbial, cardiovascular effects, ganglionic action, and acetylcholinesterase inhibition. However, it is important to note that *C. amabile* exhibits hepatotoxicity, with its toxicity being linked to its primary PAs components. Although preliminary studies suggest potential applications in the treatment of prostate diseases and alopecia, further research is needed to validate these clinical uses. Our review highlights the traditional uses, phytochemistry, biological activity, toxicity, and clinical applications of *C. amabile*. It emphasizes the essential guiding role of the indigenous medicinal knowledge system in developing new drugs. Previous studies have shown that the phytochemical and pharmacological characteristics of *C. amabile* are significantly related to its traditional medicinal practices. *Cynoglossum amabile* has excellent market potential and can be further analyzed in terms of phytochemistry, pharmacology, and toxicology, which are critical for its clinical drug safety, quality evaluation, and resource development.

## 1 Introduction

*Cynoglossum* L. (Boraginaceae) is a cosmopolitan genus comprising 75 species. In China, there are 10 species and two varieties of *Cynoglossum*, primarily distributed in regions such as Yunnan, western Guizhou, southwestern to southeastern Tibet, Western Sichuan, and southern Gansu. *Cynoglossum* is commonly found in hillsides, grasslands, marginal lands, roadsides, and the edges of coniferous forests, typically at altitudes ranging from 1250 to 4565 m above sea level (https://powo.science.kew.org/taxon/urn:lsid:ipni.org:names:30003351-2, accessed on 25 November 2023). Most of the species within the *Cynoglossum* genus are known for their richness in pyrrolizidine alkaloids (PAs), some of which are recognized as important plant hepatotoxic components (Stegelmeier, 2011; Damianakos et al., 2013; Damianakos et al., 2016). PAs also constitute the primary sources of *Cynoglossum*’s pharmacological activities (Fayed, 2021; Vrieling, 2021). Despite its medicinal potential, the value of *Cynoglossum* in traditional medicine has been largely overlooked. This is due to incidents of poisoning resulting from accidental ingestion of *Cynoglossum* or its related components (Mudge et al., 2015; Lorena et al., 2016), which have led to its classification as a harmful weed in many countries (Stegelmeier et al., 2009). However, in China, *Cynoglossum* plants hold significant ethnobotanical value and have been utilized for food, ornaments, and medicinal purposes (Xu and Zhang, 2009; Zhang et al., 2014). China boasts a wealth of *Cynoglossum* resources, with many species having a long history of use in traditional medicines among ethnic communities. A notable example is *Cynoglossum amabile* Stapf et Drumm.

*Cynoglossum amabile*, known by various names in Mandarin, such as Daotihu (倒提壶), Lanbuqun (蓝布裙), Jizhuashen (鸡爪参), Lanhuashen (蓝花参), Goushihua (狗屎花), Lanluhu (拦路虎), Yibazhua (一把抓), is a traditional medicine employed by the Buyi, Achang, Bai, Jinuo, and other ethnic groups in China (Jia and Li, 2005). According to records in Chinese Materia Medica, An Illustrated Book of Plants, Tianbao Materia Medica, and Materia Medica of Southern Yunnan, *C. amabile* is characterized by its bitter taste and cool nature. It is credited with properties such as clearing heat and dampness, alleviating fatigue, staunching bleeding, relieving cough, and providing analgesic effects. It can be administered either alone or as a component in folk traditional medical practices. When taken orally, *C. amabile* is utilized in the treatment of conditions like acute nephritis, periodontitis, hepatitis, irregular menstruation, abnormal leucorrhea, edema, acute mandibular lymphadenitis, and angina pectoris. For external applications, it is effective in the treatment of sores, boils, carbuncles, poisonous snake bites, and bruise injuries (Wu, 1963; Lan, 1975; Editorial Board of Chinese Materia Medica, Traditional Chinese Medicine Administration of China, 1998; Gong et al., 2001).

However, there is currently no up-to-date review available on the traditional utilization, phytochemistry, pharmacology, toxicology, or clinical applications of *C. amabile*. This gap in knowledge restricts the safe use of *C. amabile* in clinical medication, quality assessment, and resource development. To further promote comprehensive research, development, and utilization of *C. amabile*, this paper aims to consolidate information on its traditional use, chemical composition, pharmacological activities, toxicological effects, and clinical applications. This endeavor is intended to enhance the safety of related medications and provide a scientific foundation for the rational and correct utilization of plants with potential liver toxicity.

## 2 Methodology

Keywords, including *Cynoglossum amabile*, Daotihu, Goushihua, Lanbuqun, and Jizhuashen, were used to search related literature and data through digital resources and paper-based materials. The earliest available documents were published in 1960, while the latest was published in August 2023. These documents discussed the traditional utilization, phytochemical constituents, pharmacological activities, and safety of *C. amabile*. Much of the literature research was conducted through the following nine online scientific databases: Google Scholar, SciFinder, Web of Science, Springer Link, PubMed, Wiley, CNKI, Baidu Scholar, China Science, and Technology Journal Database. The review also included results from Ph.D. theses, M. Sc. Dissertations, online botanical databases, and relevant conference proceedings written in English and Chinese.

## 3 Botanical description and geographic distribution

**FIGURE 1:**
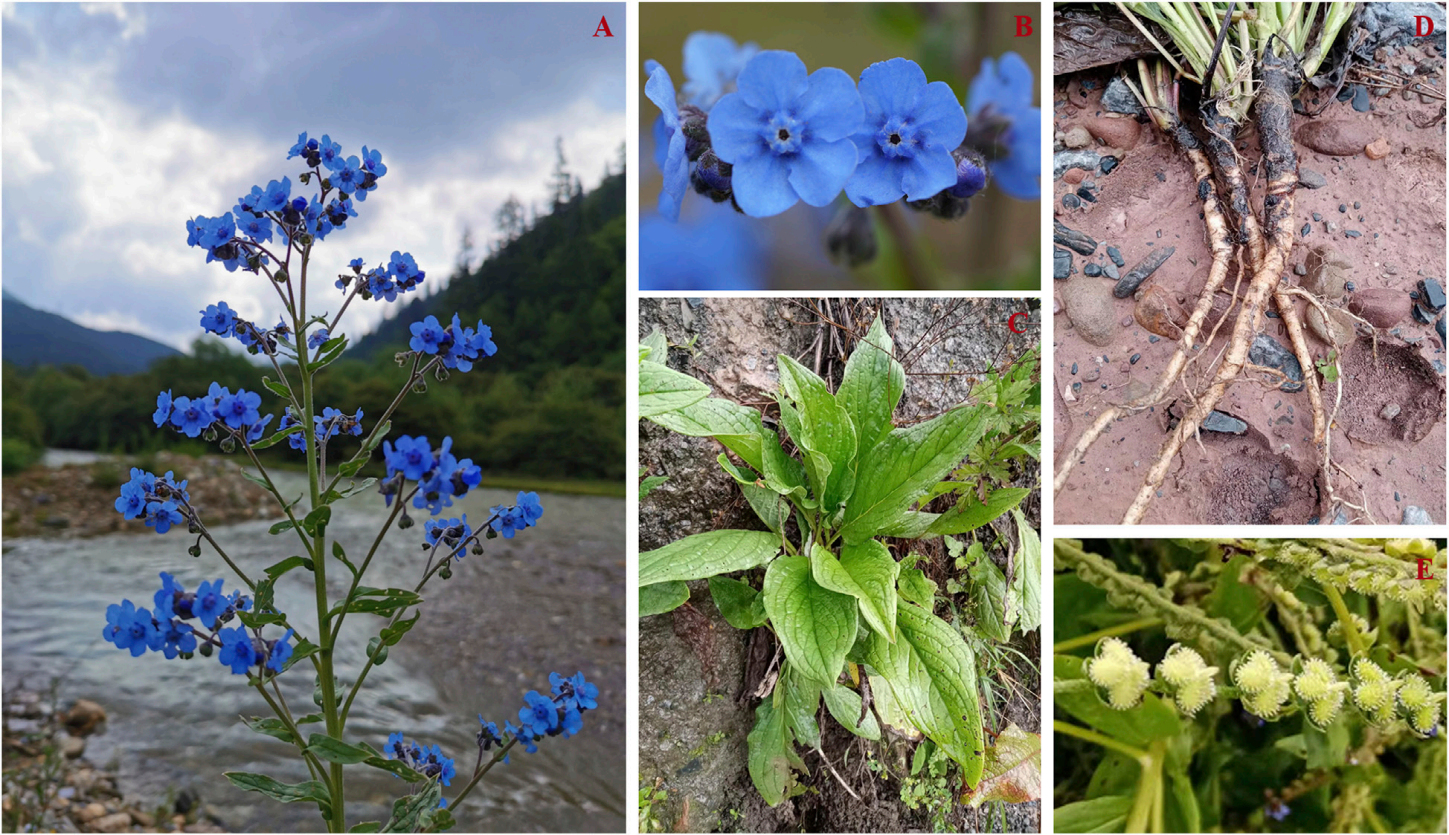
**(A)** Wild growth environment and plant morphology of *Cynoglossum amabile*. **(B)** Flowers. **(C)** Leaves. **(D)** Roots. **(E)** Seeds.

*Cynoglossum amabile* is a perennial herb that reaches a height of 15–60 cm. Its stems are either single or form several clusters, densely covered in appressed pubescence. Basal leaves have long petioles and are oblong-lanceolate or lanceolate, measuring 5–20 cm in length (including petioles) and 1.5–4 cm in width, occasionally reaching 5 cm, with dense pubescence on both surfaces. Stem leaves are oblong or lanceolate, sessile, 2–7 cm long, and exhibit very prominent lateral veins. The inflorescence is acutely branched, densely branched, extending straight upward and clustered into a panicle, without bracts. Pedicels are 2–3 mm long, slightly longer in fruit. The calyx is 2.5–3.5 mm long, densely pubescent on the outside, with ovate or oblong lobes ending in pointed tips. The corolla is typically blue with sparse white markings, measuring 5–6 mm in length and 8–10 mm in diameter at the base. Its lobes are round, about 2.5 mm long, with distinct reticulate veins, and the throat features five trapezoidal appendages, each around 1 mm in length. Filaments are approximately 0.5 mm long, inserted in the middle of the corolla tube, and the anthers are oblong, roughly 1 mm long. The style is linear-cylindrical, nearly as long as the calyx or shorter. The nutlets are ovate, 3–4 mm long, slightly concave on the back, densely covered with anchor-like spines, and have anchor-like spines along the margin at the base. They may have narrow or wide wing-like edges and a triangular insertion surface above the middle of the ventral surface (Figure 1). The flowering and fruiting period spans from May to September (source: http://www.iplant.cn/info/Cynoglossum%20amabile?t=foc, accessed on 20 July 2023).

*Cynoglossum amabile* is shade-tolerant and often blooms under conditions of sufficient sunshine. It thrives in environments with fertile soil and good drainage. The suitable temperature range for its growth is 18°C–20°C, and it is not tolerant of high temperatures. While native to China, *C. amabile* has been widely introduced in Central America and has reports of growth in northeastern North America, Kenya, and Tanzania. Nowadays, it is cultivated and naturalized in many parts of the world (source: http://www.iplant.cn/info/Cynoglossum%20amabile?t=foc, accessed on 7 August 2023).

## 4 Traditional uses

The flowers of *Cynoglossum amabile* bloom from late spring to early summer. They are blue in color and have an elegant and pleasing appearance. These flowers bear a resemblance to the “forget-me-not” (*Myosotis alpestris* F. W. Schmidt) and hold high ornamental value. They are suitable not only for combined potted plants and garden settings but also for enhancing the aesthetics of rocks, lawns, and garden pathways. In Yunnan, China, some ethnic communities cultivate *C. amabile* along fences for ornamental purposes.

In Liangshan Prefecture, Sichuan Province, China, the Yi people regard *C. amabile* as a wild edible plant and harvest its roots for food. According to Sichuan Zhong Yao Zhi, *C. amabile* possesses significant dietary therapy value. Residents in Sichuan often stew meat with the entire plant, which has a positive effect on treating conditions such as cough-induced aphonia and spitting blood (Chinese Academy of Sciences, 1960; Wang et al., 2020).

The primary value of *C. amabile* lies in its medicinal applications. The earliest records of its medicinal value can be found in “Dian Nan Ben Cao” (AD 1436), followed by mentions in ancient texts such as “Ben Cao Gang Mu Shi Yi” (AD 1765) and “Zhi Wu Ming Shi Tu Kao” (AD 1848) (Lan, 1975; Jia and Li, 2005). “Dian Nan Ben Cao” states that one name for *C. amabile* is Daotihu, also known as Yibazhua, Lanbuqun, Lanhuashen, and Goushihua, among others. It promotes urination, reduces edema, clears stomach heat, treats jaundice, relieves liver pain, and addresses seven types of hernia pain. Apart from the common blue-flowered *C. amabile*, the book mentions two other varieties with white and red flowers. The white-flowered variety was later identified as *Cynoglossum amabile* Stapf et Drumm f. *eucanthum*, and the red-flowered one as *Cynoglossum amabile* Stapf et Drumm f. *ruberum*. All three varieties can be used medicinally, with the blue-flowered one being the most commonly utilized (Lan, 1975).

Currently, many ethnic groups in China still maintain the traditional practice of using *C. amabile* for medicinal purposes (Geng et al., 2016; Gao et al., 2019; Wang et al., 2020; Zhang et al., 2022). As depicted in Table 1, different parts of *C. amabil*e, including the whole plant, roots, and leaves, are utilized for various health conditions. The entire plant is typically collected in summer, then washed and dried or used fresh. When mashed and applied externally, the fresh plant can treat traumatic bleeding, fractures, joint dislocations, carbuncle, and abscess. When the sun-dried whole plant is decocted and ingested, it has effects such as clearing heat, removing dampness, dispersing blood stasis, stopping bleeding, and relieving cough. Roots are typically gathered in autumn, washed, sliced, and dried. They are commonly employed to address conditions like bleeding due to external injury, hepatitis, hernia, dysentery, malaria, and stranguria. Leaves are effective against a range of ailments, including various types of hernia pain, small bowel pain, bladder pain, and renal cysts (Jia and Li, 2005).

**TABLE 1:** The traditional medicinal practice of Chinese ethnic groups for *Cynoglossum amabile*.

Ethnic groups	Local name	Used part	Medicinal efficacy	References
Yi	A nu de niang	Whole plant	Rheumatism, tingling in hands and feet, irregular menses, infertility	Gao et al. (2019); Jia and Li, (2005); Wang et al. (2020)
	Ta ne mo song chu bu	Root	Bleeding due to external injury, hepatitis, hernia, dysentery, malaria, stranguria, cystitis, urethritis, obstructed urinary tract, hematuria, abnormal leucorrhea	Gao et al. (2019); Jia and Li, (2005); Wang et al. (2020)
	Tu lu mu pei	Leaf	All kinds of hernia pain, small bowel pain, bladder pain, renal cyst	Gao et al. (2019); Jia and Li, (2005); Wang et al. (2020)
Lisu	Mo nai ruo	whole plant	Malaria, hepatitis, dysentery, leucorrhea, tuberculosis, cough, fracture, joint dislocation, bleeding due to external injury	Jia and Li, (2005)
Bai	Xian he zhi	whole plant	Abnormal leucorrhea, jaundice, purulent and bloody stool, ulcers and carbuncle do not collapse	Jia and Li, (2005)
	Kuan shi hu	Root	Nephritis, hepatitis, rheumarthritis, irregular menses, hernia	Jia and Li, (2005)
Naxi	Ken xian ba hai	whole plant	Lcteric hepatitis, cough caused by lung heat, whooping cough, bronchitis, dysentery, abnormal leucorrhea	Jia and Li, (2005); Zhang et al. (2022); Geng et al. (2016)
	Ba fu	Root	Nephritis, hepatitis, rheumarthritis, irregular menses, hernia	Jia and Li, (2005)
Jing po	Dvimvon byvoq	whole plant	Hepatitis, dysentery, urination pain, tuberculosis, cough, bleeding due to external injury, fracture, joint dislocation	Jia and Li, (2005)
De ang	La nu	whole plant	Hepatitis, dysentery, urination pain, tuberculosis, cough, bleeding due to external injury, fracture, joint dislocation	Jia and Li, (2005)
A chang	Hui qi gan dan	whole plant	Hepatitis, dysentery, urination pain, tuberculosis, cough, bleeding due to external injury, fracture, joint dislocation	Jia and Li, (2005)
Tibetan	Nai ma jia ma er	whole plant	Sores and boils	Jia and Li, (2005)
Ji nuo	He cha sheng niang	Root, leaf	External application to treat unknown swelling poison	Jia and Li, (2005)

## 5 Phytochemistry

Research on the chemical components of *Cynoglossum amabile* can be primarily divided into two aspects: isolation and identification. The methods for isolating and identifying these components include ethanol extraction, PLC-MS/MS, DOLPA/AOT-UPLC, and IR/MS/NMR (Culvenor and Smith, 1967; Kedzierski and Buhler, 1986; Zhang et al., 2007; Fan et al., 2016; Fan et al., 2018). Presently, a total of 47 compounds have been identified in the leaves, flowers, seeds, and whole plants of *C. amabile* (Table 2). These 47 compounds encompass alkaloids, sterols, organic acids, amino acids, saccharides, triterpenoids, fatty acids, furans, and other categories.

**TABLE 2:** Chemical constituents isolated and identified from *Cynoglossum amabile*.

No.	CAS	name	Parts of plant	Detect method	Type	References
1	17958–43-9	Amabiline	whole plant	MS/NMR	Alkaloids	Culvenor and Smith, (1967)
2	3660–62-6	Ambelline	whole plant	PLC-MS/MS		Fan et al. (2016)
3	480–83-1	Echinatine	whole plant	MS/NMR		Culvenor and Smith, (1967)
4	570–19-4	Europine	whole plant	PLC-MS/MS		Fan et al. (2016)
5	32728–78-2	Heliosupine	whole plant	DOLPA/AOT-UPLC		Fan et al. (2018)
6	520–63-8	Heliotridine	whole plant	DOLPA/AOT-UPLC		Fan et al. (2018)
7	303–33-3	Heliotrine	whole plant	DOLPA/AOT-UPLC		Fan et al. (2018)
8	303–34-4	Lasiocarpine	whole plant	DOLPA/AOT-UPLC		Fan et al. (2018)
9	6029–84-1	Rinderine	whole plant	GLC/GLC-MS		El-Shazly et al. (1996)
10	551–59-7	Supinidine	whole plant	MS/NMR		Culvenor and Smith, (1967)
11	551–58-6	Supinine	whole plant	GLC/GLC-MS		El-Shazly et al. (1996)
12	551–57-5	Viridiflorine	whole plant	PLC-MS/MS		Fan et al. (2016)
13	474–58-8	Eleutheroside A	whole plant	IR/MS/NMR	Sterols	Xu et al. (2008)
14	545–47-1	Lupeol	whole plant	NMR		Zhang, (2013)
15	83–48-7	Stigmasterol	whole plant	IR/MS/NMR		Xu et al. (2008)
16	83–46-5	*β*-sitosterol	whole plant	IR/MS/NMR		Xu and Zhang, (2009)
17	338–69-2	D-alanine	Seed	—	Organic acids	Editorial Committee of Chinese Materia Medica, Traditional Chinese medicine administration of China, (1998)
18	609–36-9	Dl-proline	Seed	—		
19	112–85-6	Docosanoic acid	whole plant	NMR		Zhang, (2013)
20	56–40-6	Glycine	Seed	—		Editorial Committee of Chinese Materia Medica, Traditional Chinese medicine administration of China, (1998)
21	6899–03-2	L-aspartic acid	Seed	—		
22	56–89-3	L-cystine	Seed	—		
23	56–86-0	L-glutamic acid	Seed	—		
24	72–19-5	L-threonine	Seed	—		
25	3588–60-1	N-carbobenzoxy-dl-leucine	Seed	—		
26	506–48-9	Octacosanoic acid	whole plant	IR/MS/NMR		Xu et al. (2008)
27	1002–84-2	Pentadecanoic acid	whole plant	NMR		Zhang, (2013)
28	99–50-3	Protocatechuic acid	whole plant	NMR		Zhang, (2013)
29	69–72-7	Salicylic acid	whole plant	NMR		Zhang, (2013)
30	6556–12-3	D-Glucuronic acid	seed	—	Saccharides	Editorial Committee of Chinese Materia Medica, Traditional Chinese medicine administration of China, (1998)
31	50–99-7	D (+)-Glucose	seed	—		
32	50–69-1	D-Ribose	seed	—		
33	58–86-6	D (+)-Xylose	seed	—		
34	63–42-3	Lactose	seed	—		
35	5328–37-0	L-Arabinose	seed	—		
35	7776–48-9	L-fructose	seed	—		
36	10030–85-0	L (+)-Rhamnose monohydrate	seed	—		
37	5989–81-1	*α*-D-Lactose monohydrate	seed	—		
38	508–02-1	Oleanolic acid	whole plant	NMR	Triterpenoids	Zhang, (2013)
40	1883–75-6	[5-(hydroxymethyl) furan-2-yl] methanol	whole plant	NMR	Furans	Xu et al. (2009)
41	17670–06-3	Delphinidin 3,5-diglucoside	Flower	—	Anthocyanins	Editorial Committee of Chinese Materia Medica, Traditional Chinese medicine administration of China, (1998)
42	17233–93-1	Viridifloric acid	whole plant	MS/NMR	Others	Culvenor and Smith, (1967)
43	—	Amabilic acid	whole plant	IR/MS/NMR		Xu et al. (2009)
44	—	2*α*,3*β*,19*α*,23-tetrahydroxyolean-12-en-28-oic acid	whole plant	IR/MS/NMR		
45	—	21-*O*-*β*-D-glucopyranosyl-2*α*,3*β*,21*β*,23-tetrahydroxyolean-12-en-28-oic acid	whole plant	IR/MS/NMR		
46	—	Benjaminamide	whole plant	IR/MS/NMR		
47	83114–74-3	7-Acetylechinatine	whole plant	GLC/GLC-MS		El-Shazly et al. (1996)

### 5.1 Alkaloids

Alkaloids are the primary constituents of *Cynoglossum*, known for their diverse pharmacological and toxicological activities, such as anti-tumor, antibacterial, anti-diabetic, and hepatotoxic effects (Berkov et al., 2020; Rasouli et al., 2020; Zhou et al., 2021). Similar to other *Cynoglossum* plants, *C. amabile* employs alkaloid accumulation as its primary chemical defense mechanism (El-Shazly et al., 2001; Frölich et al., 2007; Damianakos et al., 2016).

A total of 12 alkaloids have been isolated and identified from *C. amabile.* For instance, Culvenor and Smith utilized paper chromatography, thin-layer chromatography (TLC), and gas chromatography to analyze dried *C. amabile* (163 g). They determined that it contained 0.53% N-oxide and 0.35% free base. The total crude base (380 mg) exhibited R_F_ (paper) of 0.36, 0.40; R_F_ (TLC) of 0.10, 0.30; and R_T_ of 3.6. Subsequently, the crude base underwent countercurrent distribution between 20 mL phases of CHC_l3_ and potassium phosphate buffer at pH 7.7. After 50 transfers, base (184 mg) was isolated from tubes 20–32, with an R_F_ (TLC) of 0.10, yielding amabiline with R_F_ (paper) of 0.40 and an R_F_ (TLC) of 0.10. From tubes 38–50, base (115 mg) with an R_F_ (TLC) of 0.28 was isolated, yielding echinatine. Echinatine formed picrolonate yellow rosettes from ethanol, with a melting point of 209°C–209.5 °C (decomposition), remaining unchanged upon admixture with echinatine picrolonate (Found: C, 52.9; H, 6.0; N, 12.5. C_15_H_25_N0_5_, C_10_HgN_4_0_5_ requires C, 53.3; H, 5.9; N, 12.4%) (Culvenor and Smith, 1967). Amabiline and Echinatine are both PAs (pyrrolizidine alkaloids).

**FIGURE 2:**
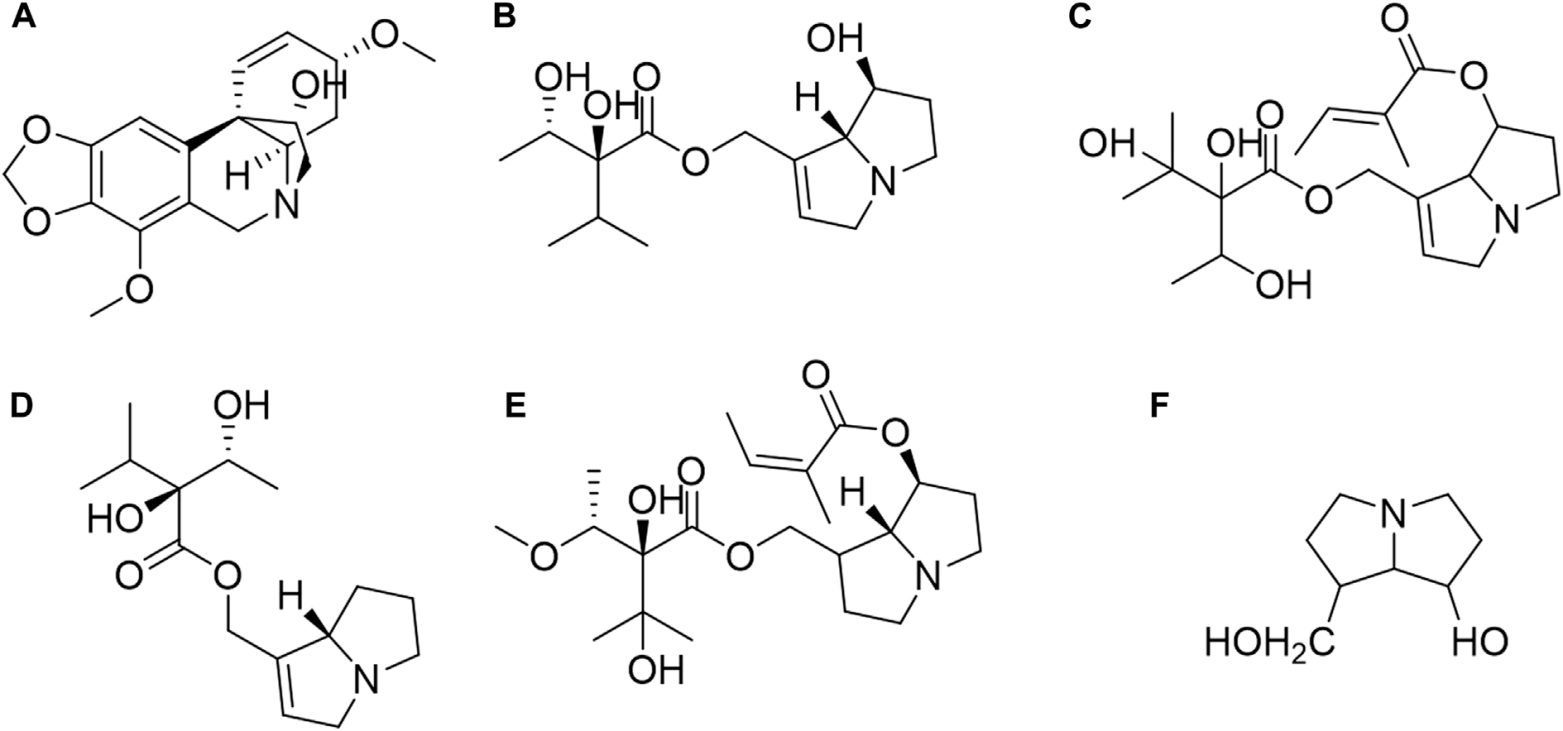
Structures of main PAs from *Cynoglossum amabile* and their basic structure. **(A)** Ambelline, **(B)** Echinatine, **(C)** Heliosupine, **(D)** Supinine, **(E)** Lasiocarpine, **(F)** The basic structure of PAs.

Additionally, the PAs in *C. amabil*e include heliosupine, supinine, and lasiocarpine. These alkaloids share a fundamental structure of 7-hydroxy-1-hydroxymethyl-6,7-dihydro-5h-pyrrolizidine, as depicted in Figure 2 (Schenk et al., 2015; Hama and Strobel, 2019). Other alkaloids found in *C. amabile* comprise heliotridine, heliotrine, rindeline, supinidine, and viridiflorine (Culvenor and Smith, 1967; El-Shazly et al., 1996; Fan et al., 2018).

### 5.2 Sterols

Sterols are steroids containing hydroxyl groups. Their structure is akin to cholesterol but with a different side chain (Zhu et al., 2009; Melo et al., 2015; He et al., 2018). Most sterols possess double bonds at C-5, with the hydroxyl group at C-3 serving as an important functional group, enabling the formation of various derivatives (Das and Kumar, 2020; Xiong et al., 2020; Yan et al., 2022). When the double bond in a sterol is saturated, it is referred to as a stanol, and when esterified, it becomes a sterol ester (Ketomäki et al., 2003; O’Neill et al., 2005). Sterols can be categorized as phytosterols, animal sterols, and fungal sterols based on their sources. Common phytosterols include sitosterol, stigmasterol, and campesterol. Cholesterol is the primary animal sterol, while ergosterol is prevalent in fungi, primarily in molds and mushrooms (Martini et al., 2021; Shi et al., 2021; Zhang et al., 2021). The diverse number of methyl groups attached to C-4, the length of the side chain on C-11, and the number and location of double bonds contribute to the greater diversity of phytosterols compared to the other two sterol types (Kritchevsky and Chen, 2005; Marangoni and Poli, 2010). Sterols have many different biological functions. For example, animal sterols can be used as components of cell membranes and constitute adrenocortical hormones and sex hormones (Goldstein et al., 2006; Matić et al., 2014). Many phytosterols also have strong pharmacological or toxicological effects, such as digitalis and ouabain, which can enhance the contraction of the myocardium and be good drugs for the treatment of heart failure (Marangoni and Poli, 2010; Garcia et al., 2015; Ravi et al., 2020).

**FIGURE 3:**
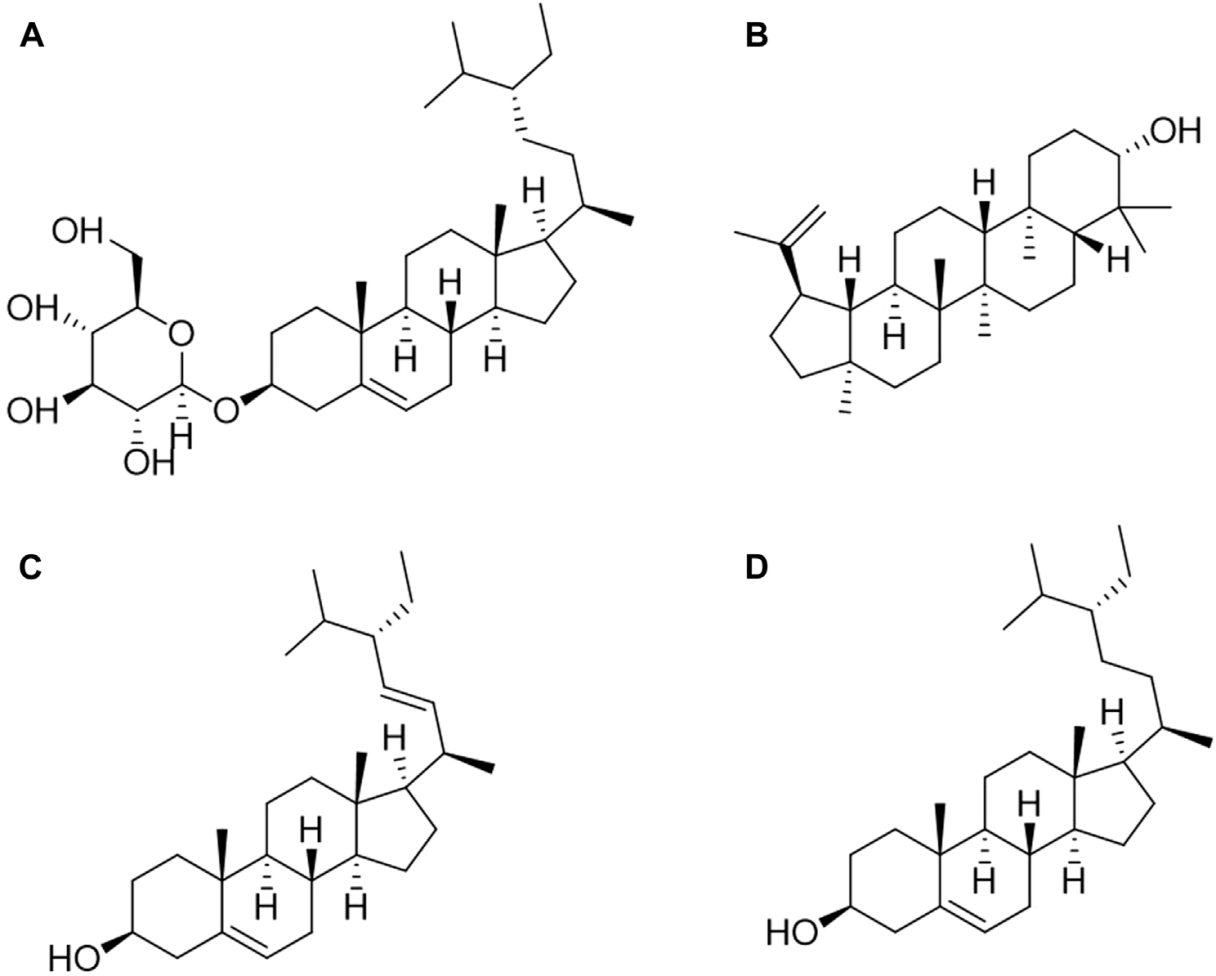
Structures of sterols from *Cynoglossum amabile*. **(A)** Eleutheroside A, **(B)** Lupeol, **(C)** Stigmasterol, **(D)** β-sitosterol.

At present, there are four sterols identified from *C. amabile*, as shown in Figure 3, which are eleutheroside A, lupeol, stigmasterol, and *β*-sitosterol (Xu et al., 2008; Xu et al., 2009; Zhang, 2013). Xu et al. processed 3 kg of dried *C. amabile* by grinding them into powder. The coarse powder underwent three reflux extractions with 95% ethanol, each lasting 2 h. The resulting extracts were combined, concentrated, and sequentially extracted with petroleum ether (PE), chloroform, and ethyl acetate (EA). The PE fraction (19 g) was mixed with 200–300 mesh silica gel and applied to a column. Elution was performed using different ratios of PE and EA in TLC. Fractions with similar characteristics were combined, underwent recrystallization purification, and Eleutheroside A (50 mg) was obtained through EA elution. Stigmasterol (40 mg) was obtained through gradient elution with PE: EA (100:3) (Xu et al., 2008). Zhang conducted a similar process involving 30 kg of dried *C. amabile*. The material was ground into powder, and 3 kg of the resulting powder was extracted using 95% ethanol reflux. The extract was dispersed in water and successively extracted with PE, chloroform, EA, and n-butanol. The PE, chloroform, and n-butanol fractions were separated and identified using various chromatographic methods, including silica gel, MCI, glucose gel LH-20, and reverse-phase silica gel Rp-18, supplemented by recrystallization for purification. In total, 12 monomeric compounds were isolated and identified. Through physical and spectral analyses, as well as TLC comparison with standard substances, compounds seven and eight were identified as lupeol (77 mg) and β-sitosterol (254 mg), respectively (Zhang, 2013).

### 5.3 Organic acids

**FIGURE 4:**
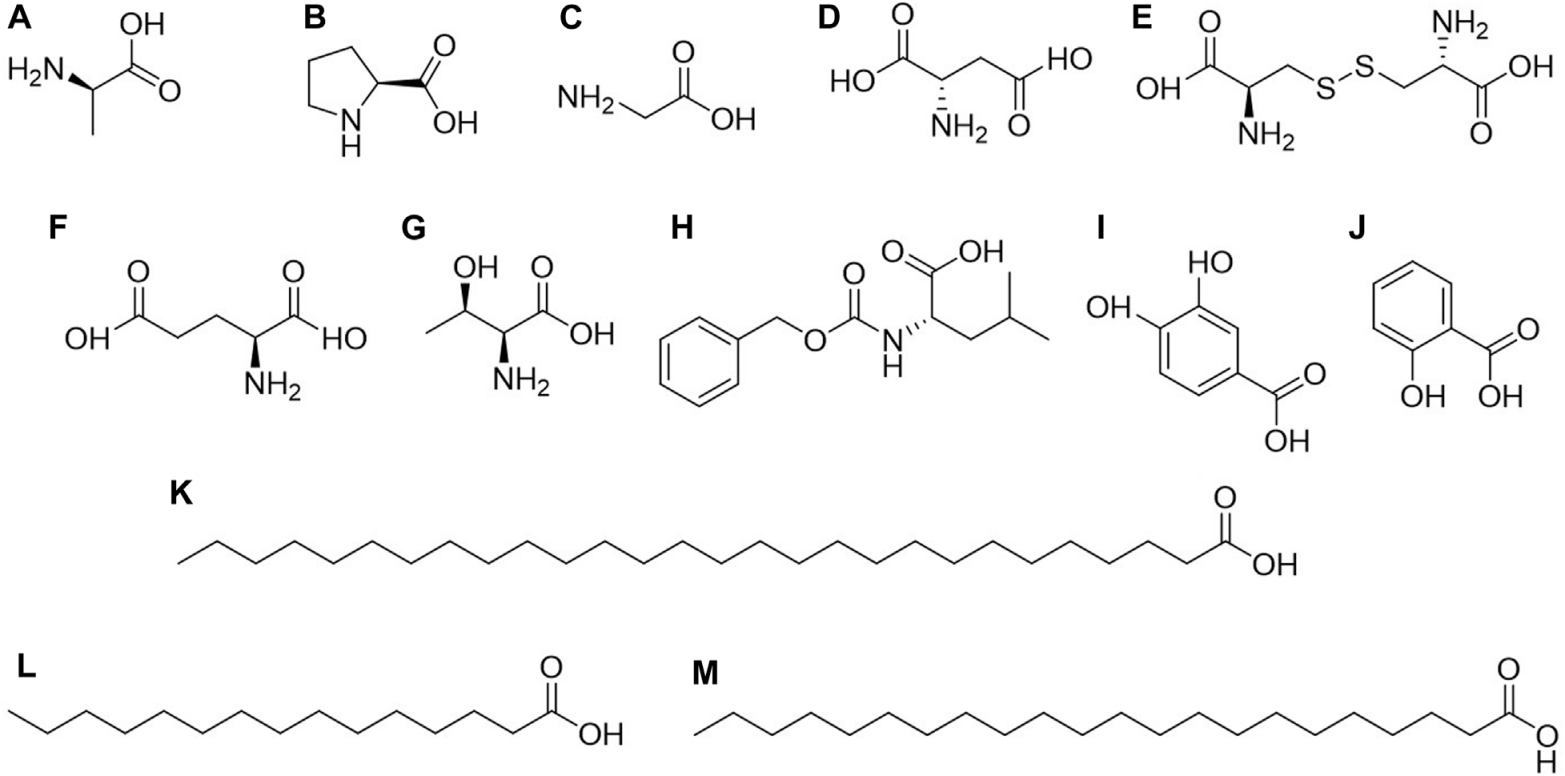
Structures of organic acids or esters from *Cynoglossum amabile*. **(A)** D-alanine, **(B)** DL proline, **(C)** Glycine, **(D)** L-aspartic acid, **(E)** L-cystine, **(F)** L-glutamic acid, **(G)** L-threonine, **(H)** N-carbobenzoxy-dl-leucine, **(I)** Protocatechuic acid, **(J)** Salicylic acid, **(K)** Octacosanoic acid, **(L)** Pentadecanoic acid, **(M)** Docosanoic acid.

Organic acids are compounds containing carboxyl groups within their molecules, encompassing carboxylic acids and their substituted counterparts. They naturally exist in animals and plants as free forms, salts, or esters (Bae and Lee, 2015; Ghislain et al., 2021; Huang et al., 2021). In a broader context, organic acids cover a wide range of compounds, including fatty acids or esters, phenolic acids or esters, amino acids or esters, and more. According to reported compounds, *C. amabile* contains 13 types of organic acids, including amino acids, phenolic acids, and fatty acids (Figure 4).

Amino acids are primarily found in the seeds of *C. amabile*, including D-alanine, DL proline, Glycine, L-aspartic acid, L-cystine, L-glutamic acid, L-threonine, and N-carbobenzoxy-dl-leucine (Editorial Committee of Chinese Materia Medica, Traditional Chinese Medicine Administration of China, 1998), while phenolic acids and fatty acids are present throughout the entire plant. Among fatty acids, three types are identified: docosanoic acid, pentadecanoic acid, and octacosanoic acid. Salicylic acid and protocatechuic acid are the primary phenolic acids. As mentioned above, the components found in *C. amabile* by Xu et al. also include octacosanoic acid (200 mg). The 12 monomeric compounds separated and extracted from *C. amabile* by Zhang include docosanoic acid (11 mg), pentadecanoic acid (23 mg), and protocatechuic acid (18 mg) (Xu et al., 2008; Zhang, 2013). It is worth noting that these compounds are essential in organic synthesis and have widespread applications in pharmaceuticals, pesticides, rubber, dyes, food, and the fragrance industry, particularly in daily chemical products (Spepi et al., 2016; Tan et al., 2020).

### 5.4 Saccharides and others

**FIGURE 5:**
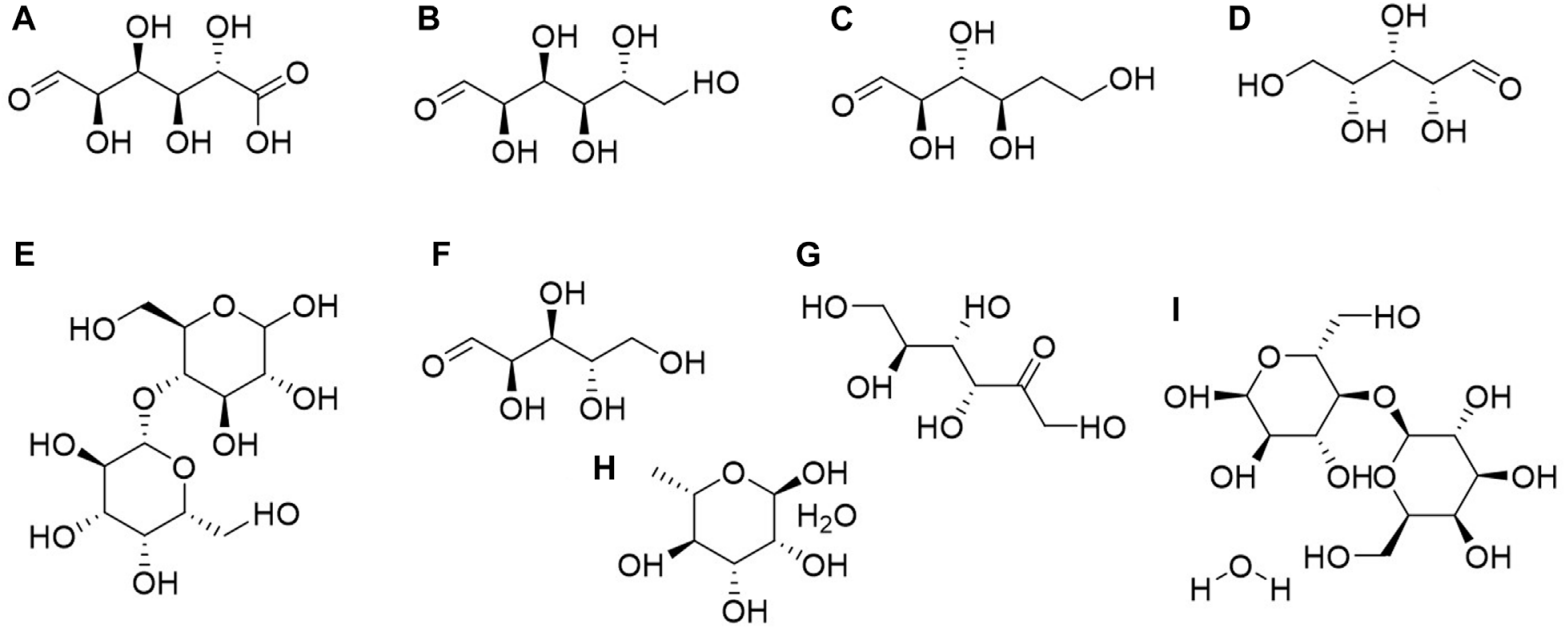
Structures of saccharides from the seeds of *Cynoglossum amabile*. **(A)** D-Glucuronic acid, **(B)** D (+)-Glucose, **(C)** D-Ribose, **(D)** D (+)-Xylose, **(E)** Lactose, **(F)** L-Arabinose, **(G)** L-fructose, **(H)** L (+)-Rhamnose monohydrate, **(I)** α-D-Lactose monohydrate.

Structurally, saccharides are polyhydroxy aldehydes and ketones and their polymers. They are widely present in plants, serving as vital components with significant roles in plant physiological and biochemical processes (Kapaev and Toukach, 2018; Xue et al., 2018; Li et al., 2019). Based on their structural differences, saccharides can be categorized into monosaccharides (such as glucose, fructose, and galactose), disaccharides (including sucrose, maltose, and lactose), and polysaccharides (such as starch and glycogen). Currently, nine saccharides have been isolated and identified from *C. amabile* seeds, encompassing both monosaccharides and polysaccharides (Editorial Board of Chinese Materia Medica, Traditional Chinese Medicine Administration of China, 1998; Alee et al., 2021; Debras et al., 2021) (Figure 5).

Furthermore, *C. amabile* contains triterpenoid components, such as oleanolic acid, furan components [5- (hydroxymethyl) furan-2-yl] methanol in the entire plant, anthocyanin components like delphinidin 3,5-diglucoside in its flowers, and various other components, including viridifloric acid, amabilic acid, and 2α,3β,19α,23-tetrahydroxyolean-12-en-28-oic acid. Among these, oleanolic acid stands out as an intermediate compound used in medicine, pesticides, and dyes, with wide applications in the pharmaceutical and cosmetic industries (Culvenor and Smith, 1967; El-Shazly et al., 1996; Xu et al., 2009; Zhang, 2013).

## 6 Pharmacology

*Cynoglossum amabile* is a well-known traditional Chinese medicine often utilized in the treatment of hepatitis, dysentery, cough caused by tuberculosis, bleeding due to trauma, abnormal leucorrhea, and other diseases. As previously mentioned, *C. amabile* contains alkaloids, sterols, organic acids, saccharides, triterpenoids, and various other chemical components. Its primary component is PAs, indicating that this plant holds potential as an ethnomedicine deserving of significant research. A summary of the biological and pharmacological activities associated with *C. amabile* is presented in Table 3. Detailed information can be found below.

**TABLE 3:** Summary of the pharmacological activities of *Cynoglossum amabile*.

Pharmacological activity	Tested substance/part	Tested system/organ/cell	Tested dose	Results	Refs
Anti-inflammatory	Amabilic acid	SD mice	Amabilic acid high dose 60 mg/kg, amabilic acid low dose 30 mg/kg	Amabilic acid had an obvious inhibitory effect on mouse auricle swelling induced by xylene (*p* < 0.01), and the effect was better than aspirin	Xu et al. (2009)
	Echinatine	BM-derived macrophages (BMDMs), LPS-primed human peripheral blood mononuclear cells (hPBMCs), NLRP3 inflammasome activation in mice	Mice that received 120 mg/kg dose of echinatin (3 times higher than the dose used for the septic shock experiments) daily for 15 days	Echinatin inhibits nigericin-induced NLRP3 inflammasome activation in both mouse and human immune cells.	Xu et al. (2021)
Anti-tumor	Echinatine, europine, heliotrine, lasiocarpine, supinine	Adenocarcinoma 755 in mice, lymphoid leukemia L1210, sarcoma 180, Walker 256 (intramuscular) in rats, Walker 256 (subcutaneous) in rats, and plasmacytoma 1 in hamsters	25% increase in survival time for leukemia 1210% and 50% inhibition of kB cell culture at a concentration of 1 mcg/mL	Tumor-inhibitory activity is widely exhibited among PAs	Culvenor and Smith, (1967)
Antimicrobial	Four different polarities of petroleum ether phase, chloroform phase, ethyl acetate phase and water phase of the *C. amabile*’s aerial part ethanol extract	Five bacteria including *Escherichia coli*, *Bacillus subtilis*, *Proteus vulgaris*, *Staphylococcus aureus* and *Pseudomonas aeruginosa*, and five plant pathogenic fungi including *Botryosphaeria rhodina*, *Fusarium oxysporum*, *Fusarium solani*, *Corynespora cassiicola* and *Colletotrichum acutatum*	*E. coli*: 7.7*10^6^ CFU/mL, *B. subtilis*: 3.5*10^6^ CFU/mL, *P. vulgaris*: 1.9*10^6^ CFU/mL, *P. aeruginosa*: 5.5*10^6^ CFU/mL, *S. aureus*: 4.8 * 10^6^ CFU/mL, aqueous phase (0.19 g/mL), ethyl acetate phase (0.04 g/mL), petroleum ether phase (0.28 g/mL) and chloroform phase (0.037 g/mL)	*C. amabile* has certain antibacterial activity, but its inhibitory effect on various strains is different	Wang et al. (2014)
Against ischemia/reperfusion heart	Echinatine	Sprague–Dawley rats	Echinatin (0.5 and 2.5 μg/mL)	Echinatin limited the contents of CK and LDH, improved the left ventricular developed pressure, reduced the contents of malondialdehyde, interleukin-6, and tumor necrosis factor-α, and increased the superoxide dismutase activity	Tian, et al. (2016)
Ganglionic blocking action	Heliotrine	Adult mongrel dogs (8–15 kg), adult guinea pigs of either sex (500g–1 kg), albino mice of either sex (15–30 g)	Acute toxicity (24 h mortality) was studied by injecting graded doses of heliotrine (100, 200, 300, 400 and 500 mg/kg) intraperitoneally in groups (10 per each) of albino mice of either sex (15–30 g)	*S*tudies with heliotrine revealed that it selectively reduces the autonomic ganglion stimulant effects of nicotine, suggesting that it possesses ganglion blocking activity	Pandey et al. (1982)
Acetylcholinesterase inhibitory activity	Heliosupine, heliotrine	Acetylcholine esterase (AChE), butyrylcholinesterase (BChE), choline acetyltransferase (ChAT), and neuroreceptors, such as α1- and α2-adrenergic, nicotinergic (nACh), muscarinergic (mACh) and serotonin2 (5-HT2) receptors	The membrane preparation was incubated for 40 min with differing concentrations of PA or 1 mM nicotine as a positive control	They show significant binding activities to mACh and 5-HT2 receptors, IC_50_ values were between 8.7 μM and 512.5 μM	Schmeller et al. (1997)
Aphicidal activities	Amabiline	Potter spray tower assay, *Aphis citricola*	Put the plate under a Potter tower and sprayed at 78 kPa with 2 mL of each concentration	Amabiline exhibited considerable aphicidal activity with LD_50_ values of 67.44 ng/aphid	Yan et al. (2018)

### 6.1 Anti-inflammatory

To assess the anti-inflammatory activity of amabilic acid extracted from *C. amabile*, SD mice were used as the test subjects. Aspirin served as the positive control, with a dosage of 60 mg/kg in the high-dose amabilic acid group and 30 mg/kg in the low-dose group. After 5 days of continuous intragastric administration, it was observed that amabilic acid significantly inhibited mouse auricle swelling caused by xylene (*p* < 0.01), surpassing the performance of aspirin, even when administered at half the dosage of aspirin (Xu et al., 2009). Therefore, it is essential to select an appropriate pharmacological model for further compound studies.

Echinatine, a primary component of PAs in *C. amabile*, was employed to test its inhibitory effect on NLRP3 inflammasome activation in mouse BM-derived macrophages (BMDMs) and LPS-primed human peripheral blood mononuclear cells (hPBMCs). The study revealed that echinatin exhibited no cytotoxicity at doses below 100 μM in BMDMs but effectively inhibited NLRP3 inflammasome by binding to heat-shock protein 90 (HSP90), thereby inhibiting its ATPase activity and disrupting the association between the cochaperone SGT1 and HSP90-NLRP3. Additionally, echinatin potently suppressed caspase-1 activation and IL-1β secretion in response to canonical NLRP3 stimuli. Furthermore, it demonstrated favorable pharmacological effects on liver inflammation and fibrosis in a mouse model of nonalcoholic steatohepatitis (NASH). These findings suggest that echinatin is a broad inhibitor of NLRP3 inflammasome, capable of suppressing both canonical and noncanonical NLRP3 inflammasome activation (Xu et al., 2021). This implies that *C. amabile* may serve as a promising candidate for therapeutic interventions in NLRP3 inflammasome-driven diseases.

### 6.2 Anti-tumor

Following the Cancer Chemotherapy National Service Center (CCNSC) protocols, the tumor-inhibitory properties of echinatine, europine, heliotrine, lasiocarpine, and supinine, the primary components in *C. amabile*, were assessed. Several tumor models including adenocarcinoma 755 in mice, lymphoid leukemia L1210, sarcoma 180, Walker 256 (intramuscular) in rats, Walker 256 (subcutaneous) in rats, and plasmacytoma one in hamsters were employed. Various dosing regimens were applied, ranging from one dose daily for 11 days to one dose daily for 15 days.

The results revealed widespread tumor-inhibitory activity among PAs. The tumors most frequently inhibited were Walker 256 (intramuscular) and adenocarcinoma 755. In some cases, the activity level against the former was very high, requiring heliotrine at a dose level of 125 mg/kg for complete or near-complete tumor destruction. Both heliotrine and lasiocarpine displayed activity against sarcoma 180. Lasiocarpine was effective against Walker 256 (subcutaneous) tumors but showed limited activity against leukemia 1210 and kB cell culture. Heliotrine exhibited strong activity against Walker 256 (intramuscular), while lasiocarpine showed moderate activity, and echinatine and supinine were essentially inactive. Although the known acute and chronic effects of PAs do not favor the development of tumor-suppressive drugs in analogs, these results suggest that for tumor-inhibitory activity, an optimum level or set of levels exists for one or more of the interrelated properties, such as water solubility, lipid-water partition coefficient, and base strength (Culvenor and Smith, 1967). Therefore, it may be possible to use these components to inhibit mitosis selectively, durably, and completely in parenchymal hepatocytes. If this activity can extend to liver cell-derived tumor cells, it holds promise for complete liver cancer inhibition and benefits the liver.

### 6.3 Antimicrobial

Four different polar fractions of the ethanol extract of *C. amabile*’s aerial parts were used to investigate their inhibitory effects on five bacteria, including *Escherichia coli*, *Bacillus subtilis*, *Proteus vulgaris*, *Staphylococcus aureus*, and *Pseudomonas aeruginosa*, as well as five plant pathogenic fungi, including *Botryosphaeria rhodina*, *Fusarium oxysporum*, *Fusarium solani*, *Corynespora cassiicola*, and *Colletotrichum acutatum*. The results demonstrated that the chloroform fraction had an inhibitory effect on *E. coli* and *P. vulgaris*, with a minimum inhibitory concentration (MIC) of 3 mg/mL. The EA fraction displayed inhibitory effects on *E. coli*, *B. subtilis*, and *P. vulgaris*, with an MIC of 2 mg/mL. The aqueous fraction only exhibited an inhibitory effect on *E. coli*, with an MIC of 20 mg/mL. The chloroform fraction displayed the most potent inhibitory effect on *Fusarium solani*, with a concentration of 50% of maximal effect (EC_50_) of 1.48 mg/mL. The EA fraction demonstrated the most potent inhibitory effects on *B. rhodina* and *C. cassiicola*, with EC_50_ values of 1.78 mg/mL and 0.63 mg/mL, respectively (Wang et al., 2014). These findings indicate that *C. amabile* possesses certain antibacterial activity, although its inhibitory effect varies among different strains.

However, this experiment only preliminarily conducted *in vitro* testing of *C. amabile*’s whole plant. Further research is needed to understand the mechanism of action on bacteria and plant pathogenic fungi through *in vivo* testing, separation and purification of effective antibacterial components from different parts of the plant, and the assessment of changes in the proportion of antibacterial and bacteria-promoting active substances.

### 6.4 Cardiovascular effect

To investigate the protective effect of echinatine on myocardial ischemia/reperfusion (I/R) injury in rats, adult Sprague-Dawley rats were randomly divided into five groups. Myocardial infarct size was estimated through 2,3,5-triphenyltetrazolium chloride staining. The coronary effluent was analyzed for the release of lactate dehydrogenase (LDH) and creatine kinase (CK) to assess cardiac injury. Concentrations of malondialdehyde (MDA), interleukin-6 (IL-6), and tumor necrosis factor-α (TNF-α) were determined, along with superoxide dismutase (SOD) activity using ELISA. Additionally, cardiomyocyte apoptosis analysis was conducted with POD, an *in-situ* cell death detection kit. The results indicated that echinatin (0.5 and 2.5 μg/mL) pretreatment enhanced the maximum up/down rate of the left ventricular pressure (±dp/dtmax), improved heart rate, increased left ventricular developed pressure (LVDP), enhanced coronary flow, and reduced CK and LDH levels in the coronary flow of the treated group compared to the I/R group. Echinatine limited CK and LDH levels, improved LVDP, reduced MDA, IL-6, and TNF-α levels, and increased SOD activity. The infarct size and cell apoptosis in the hearts of the rats in the echinatin-treated group were smaller and lower, respectively, than those in the hearts of the rats in the I/R control group. Therefore, echinatin exhibits a protective effect against I/R-induced myocardial injury (Tian, et al., 2016).

This effect of echinatine may be attributed to its antioxidant and anti-inflammatory activities. Researchers have reached a consensus that oxidative stress can trigger significant myocardial injury after I/R through certain pathways (Nazari et al., 2015; Sun et al., 2015). In this experiment, echinatine treatment significantly reduced the MDA content in I/R rats and improved the I/R-induced deterioration of total antioxidant capacity (SOD activity). Inflammation, which plays a crucial role in many disease states, is associated with enhanced expression of adhesive molecules in the vasculature, leading to the infiltration of larger populations of neutrophils and monocytes/macrophages. The release of pro-inflammatory cytokines from these activated leukocytes can cause tissue damage (Varela et al., 2013). The results of this experiment showed that I/R increased the production of these cytokines, while echinatine treatment reduced their concentrations. Overall, this extract may inhibit myocardial I/R injury through its anti-inflammatory activities.

### 6.5 Ganglionic blocking action

Heliotrine is one of the primary PAs found in *C. amabile*. To test its ganglionic blocking action, adult mongrel dogs (8–15 kg) of either sex were anesthetized with chloralose (70 mg/kg, i. v.). Drugs were administered through the cannulated femoral vein. The effect of graded doses of the test drug on blood pressure and nictitating membrane responses to nicotine (25 μg/kg) and adrenaline (10 μg/kg) was recorded. An interval of 5–10 min was maintained between two successive drug administrations. Meanwhile, the ED_50_ of the test drug against nicotine-induced spasmogenic response was determined on isolated guinea pig ileum. The results were compared with pentolinium, a known ganglion blocker. *In vivo* and *in vitro* experiments with heliotrine revealed that it selectively reduces the autonomic ganglion stimulant effects of nicotine, suggesting that it possesses ganglion blocking activity. *In vitro* studies suggest that heliotrine is approximately 13 times less potent than pentolinium. Additionally, heliotrine has neither central nervous system activity nor central muscle blocking effects and does not improve spasms caused by acetylcholine and barium chloride. The potentiation of adrenaline-induced vasopressor and nictitating membrane responses by the test compound may be caused by decentralization supersensitivity, a phenomenon known to occur after the administration of ganglion-blocking agents (Pandey et al., 1982).

### 6.6 Other crucial activities

Studies have indicated that cholinergic neurons in the basal forebrain of patients with Alzheimer’s disease (AD) are absent, the activity of acetylcholinesterase (AChE) is increased, and the content of acetylcholine, a neurotransmitter, is decreased. Consequently, targeting AChE and inhibiting its activity is a classic strategy for treating AD (Ibach and Haen, 2004; Kou et al., 2021). To analyze the interaction of PAs, such as heliosupine and heliotrine, with acetylcholine-related enzymes (acetylcholinesterase and butyrylcholinesterase), choline acetyltransferase (ChAT), and neuroreceptors (α1-and α2-adrenergic, nicotinergic [nACh], muscarinergic [mACh], and serotonin2 [5-HT2] receptors), testing was conducted. The results indicated that heliosupine and heliotrine displayed significant binding activities to mACh and 5-HT2 receptors, with IC_50_ values of 390.0 μM and 52.2 μM, 77.1 μM and 535.4 μM, respectively. This suggests that *C. amabile* may hold potential as a therapeutic agent for AD.

Furthermore, in an aphid resistance activity test experiment, amabiline was tested against Aphis citricola. The results demonstrated that amabiline exhibited considerable aphicidal activity, with LD_50_ values of 67.44 ng/aphid (Yan et al., 2018). Aphids are among the most destructive and economically significant plant pests globally, causing substantial financial losses. These results suggest that *C. amabile* has the potential to be developed as a biological pesticide to combat the harm caused by aphids.

## 7 Toxicity

Since the 1930s, there have been explosive cases or individual cases of human liver poisoning caused by plants containing PAs in Jamaica, India, Afghanistan, the United States, Europe, and other places, leading to global concern about its hepatotoxicity (Mathon et al., 2014; Yang et al., 2018; Xu et al., 2019). Modern research indicates that the most basic structural unit of PAs’s toxicity is when there are double bonds at one and 2, and at least one hydroxyl group at seven or nine is esterified. The toxicity of diesters is greater than that of monoesters, and macrocyclic diester PAs are more toxic than open-chain diesters (Hartmann et al., 1997; Fu et al., 2014). The pharmacological activity and toxicity of *Cynoglossum amabile* are derived from a large number of PAs (Yan et al., 2011; Vrieling, 2021).

### 7.1 Hepatic and pulmonary toxicity

Numerous experiments and clinical observations have revealed that the toxicity of PAs often occurs in the liver. Exposure to large amounts can lead to acute poisoning, characterized by hepatic veno-occlusive disease (HVOD) or hepatic sinusoidal obstruction syndrome (SOS). Long-term ingestion of small amounts of PAs results in chronic toxicity, manifested as hepatic giant cell formation and hepatic fibrosis (Tang et al., 2007; Tang and Hattori, 2011). PAs undergo metabolic activation by liver P450s, producing pyrrolic metabolites or “metabolic pyrroles.” These pyrrolic metabolites enter the cytoplasm, where they react with essential life substances such as thiol-containing amino acids, small peptides, proteins, enzymes, and RNA. Another portion degrades rapidly, forming a series of secondary toxic metabolites, including tissue-bound pyrroles, glutathione pyrrolic conjugates (e.g., 7-GSH-DHR), and dehydroretronecine (DHR). These reactions lead to continuous poisoning of liver cells during detoxification, resulting in irreversible liver cell dysfunction, necrosis, and tissue damage (Han et al., 2011; Yang et al., 2017; Suparmi et al., 2020).

The lungs are another important target organ for the toxicity of certain PAs, manifesting as pulmonary arterial hypertension and pulmonary veno-occlusive disease. The formation of pyrrolic metabolites is also implicated in lung toxicity. For example, *in vitro* experiments show that PAS in rats generates pyrrolic-protein adducts in the liver and lungs. Severe damage occurs in the pulmonary artery endothelial cells and abnormal proliferation of pulmonary artery smooth muscle cells when mild liver damage is present in rats. These results suggest that a significant portion of dehydropyrrolizidine alkaloids (DHPAs) transfers from the liver to the lungs, preferentially inducing lung toxicity (Kempf et al., 2010; Sun et al., 2019; Song et al., 2020). However, the mechanism behind pulmonary toxicity, whether it is related to liver metabolism, and how pyrrolic metabolites are transported from the liver to the lungs remain unclear (Tang and Hattori, 2011; Nakai et al., 2019).

### 7.2 Genotoxicity and carcinogenicity

Genotoxicity refers to the ability of a substance to damage cellular DNA directly or indirectly, causing gene mutations or mutations in the body, with the potential or tendency to induce mutations and carcinogenesis. Genotoxic substances have the characteristic of causing damage to genetic material even at low concentrations and are highly toxic (Tang and Hattori, 2011; Luckert et al., 2015). After metabolic activation, PAs exhibit various genetic toxicities. Long-term ingestion of PAs leads to changes in cell morphology, enlargement of cell nuclei, loss of cell metabolic function, mitotic inhibition, lipid degeneration, and cell carcinogenesis. This includes DNA binding, DNA cross-linking, DNA-protein cross-linking, and chromosomal aberrations (Xia et al., 2008; Brown et al., 2015). For example, treatment of P53 knockout mice with different concentrations of PAs shows dose-dependent promotion of tumor occurrence and development, primarily manifested as hepatic hemangiosarcoma with accompanying vascular dilation (hepatic peliosis) (Brown et al., 2015).

The carcinogenic effect of PAs is related to the formation of pyrrolic-DNA adducts. PAs and PANOs in rat livers produce pyrrolic derivatives that bind to DNA, generating pyrrolic-DNA adducts, potential biomarkers for PAs’ carcinogenicity. The levels of adduct formation differ for PAs with different structures, with the ranking as follows: diester PAs > monoester PAs - diester PANOs >> non-esterified (Xia et al., 2008).

### 7.3 Toxicity control methods

Due to the complex chemical structure of PAs, removing these components poses significant challenges, a problem faced by many herbal medicines containing PAs. Currently, there is no universally accepted and determined method to completely remove pyrrolizidine alkaloids (PAs) from plants. However, research indicates that PAs are primarily eliminated through hydrolysis reactions and N-oxidation excretion (Habs et al., 2018). When exposure to PAs is low, reduced glutathione can bind with the dehydrogenated metabolites of PAs, preventing the latter from forming toxic complexes with DNA or proteins, representing the main detoxification pathway for PAs (Yang et al., 2016). In addition, oxidative damage also contributes to the toxicity of PAs (Yang et al., 2016; Habs et al., 2018), so substances with antioxidant properties also have a certain protective and ameliorative effect on the toxicity of PAs.

Currently, many countries or organizations have established regulations regarding the widespread presence and harm of PAs in human food and natural medicines (Kempf et al., 2010). For example, the World Health Organization (WHO) has recommended monitoring the content of PAs in honey and dairy products and issued health and safety guidelines on pyrrolizidine alkaloids in 1989. Dutch authorities stipulate that the total PAs content in 1 kg or 1 L of herbal products or extracts must not exceed 1 μg (1 ppb) (Kempf et al., 2010). The U.S. Food and Drug Administration (FDA) prohibits the use of comfrey (Symphytum) plants from the Boraginaceae family in the food processing industry (Tang and Hattori, 2011). Recently, the European Commission has mandated that pyrrolizidine alkaloid components should not be detectable in borage oil (primarily seed oil from *Echium plantagineum* L. of the Boraginaceae family) used as a food ingredient or additive (with a detection limit of 4 ppb) (Kempf et al., 2010; Tang and Hattori, 2011; Luckert et al., 2015). These regulations are worth considering and learning from when applying and promoting the use of herbal materials containing PAs, such as *C. amabile*, *Onosma paniculatum* Bur. Et Franch, and *Senecio scandens* Buch.-Ham. ex D. Don.

## 8 Clinical application

In traditional medicine, *Cynoglossum amabile* is commonly used by people in Southwest China to treat malaria, hepatitis, dysentery, urinary pain, leucorrhea, tuberculosis, and cough. It is also applied externally to treat trauma, bleeding, fractures, and joint dislocations. The researchers are currently actively exploring the clinical applications of this herbal medicine.

In the treatment of cardiovascular diseases, Xinyihao, a compound preparation composed of *C. amabile*, possesses functions such as promoting blood circulation, removing blood stasis, strengthening the spleen and Qi, and reducing swelling and pain. It can increase coronary flow, resist coronary spasms caused by pituitrin, reduce oxygen consumption, and withstand atmospheric and low-pressure hypoxia (Dai, 2015; Li, 2017).

According to a Chinese patent report, using *C. amabile* and its extracts to treat prostate disease and sexual dysfunction caused by prostate disease offers fast and reliable effects. They can enhance and repair certain physiological functions of the human body, improving overall defense and quality of life (Zhao, 2007a).

*Cynoglossum amabile* extract is used to treat hemorrhoids, constipation, anal prolapse, and other anorectal diseases. It exhibits rapid onset, a stable effect, improvement and repair of human physiological functions, and increased resistance. It has been used by nearly 100 patients with an effective rate of about 90%, offering a new option for clinical treatment of these conditions (Zhao, 2016).

Additionally, preparations made from *C. amabile*’s original medicinal materials, processed products, or extracts have shown a positive effect in treating alopecia, with an effective rate of about 85%, especially in cases of androgenic alopecia. After 30 days of treatment, patients generally experience reduced hair loss. After 2–3 months of use, atrophic hair follicles begin to repair, new hair grows in bald areas, and the bald area is gradually covered by new hair. Hair growth and metabolism gradually return to normal (Zhao, 2007b).

However, as a toxic plant medicine, there is a far from sufficient understanding of the clinical research on the use of *C. amabile*. Limited patent reports only indicate the potential future applications of this medicine and provide basic formulations and preparation methods, without offering detailed clinical research results and relevant parameters. For the promotion and application of a medicine, specialized clinical studies are indispensable. This includes specifying the types of clinical studies conducted (open-label, blind, controlled trials, *etc.*), dosage administration (and whether the extract is standardized), duration, and study outcomes (the percentage of subjects showing a positive response to the treatment). Other crucial information involves the extract/compound used, parameters influenced by the extract/compound administration, types of compounds that may have therapeutic potential and their mechanisms of action, and whether registered subjects reported any side effects or toxicity.

## 9 Conclusion

*Cynoglossum amabile* is commonly used in ethnomedicine in Southwest China and has a long history of medicinal use. This paper provides a comprehensive review of the botanical description, geographic distribution, traditional utilization, phytochemistry, pharmacology, toxicology, and clinical applications of *C. amabile*. The whole plant, roots, and leaves of *C. amabile* are often utilized as medicine in folk practices, each exhibiting different efficacies. Phytochemical studies have identified the main chemical components in *C. amabile* as pyrrolizidine alkaloids (PAs), sterols, and organic acids or esters. Among these, PAs are the most abundant, and the pharmacological effects, including anti-inflammatory, anti-tumor, anti-microbial, cardiovascular effects, ganglionic blocking, acetylcholinesterase inhibition, and hepatotoxicity of *C. amabile*, are primarily attributed to them. The absorption, distribution, metabolism, and excretion of PAs are closely linked to their pharmacological effects and toxicity mechanisms.

Despite its limited application in clinical settings, there are numerous research gaps concerning *C. amabile*. It is crucial to analyze this plant using innovative technologies and methodologies. For instance, there is insufficient research on the *in vivo* and *ex vivo* metabolism of PAs in *C. amabile*, particularly regarding their pharmacokinetics (absorption, distribution, and excretion) in animals and humans. Pharmacokinetic studies can unveil the metabolic pathways and biological activities of the identified components or differential metabolites in the root, stem, leaf, flower, seed, and other parts of *C. amabile*, thereby establishing the scientific foundation for its traditional folk use and offering guidance for further development and application. Moreover, the presence of salicylic acid, protocatechuic acid, and oleanolic acid in *C. amabile* suggests its significant potential in the daily chemical industry. However, it is essential to prioritize an in-depth analysis of the chemical components.
